# DeconPeaker, a Deconvolution Model to Identify Cell Types Based on Chromatin Accessibility in ATAC-Seq Data of Mixture Samples

**DOI:** 10.3389/fgene.2020.00392

**Published:** 2020-06-08

**Authors:** Huamei Li, Amit Sharma, Kun Luo, Zhaohui S. Qin, Xiao Sun, Hongde Liu

**Affiliations:** ^1^State Key Laboratory of Bioelectronics, School of Biological Science and Medical Engineering, Southeast University, Nanjing, China; ^2^Department of Ophthalmology, University Hospital Bonn, Bonn, Germany; ^3^Department of Neurosurgery, Xinjiang Evidence-Based Medicine Research Institute, First Affiliated Hospital of Xinjiang Medical University, Ürümqi, China; ^4^Department of Biostatistics and Bioinformatics, Rollins School of Public Health, Emory University, Atlanta, GA, United States

**Keywords:** chromatin accessibility, cell type, deconvolution, mixture samples, gene expression

## Abstract

While our understanding of cellular and molecular processes has grown exponentially, issues related to the cell microenvironment and cellular heterogeneity have sparked a new debate concerning the cell identity. Cell composition (chromatin and nuclear architecture) poses a strong risk for dynamic changes in the diseased condition. Since chromatin accessibility patterns play a major role in human diseases, it is therefore anticipated that a deconvolution tool based on open chromatin data will provide better performance in identifying cell composition. Herein, we have designed the deconvolution tool “DeconPeaker,” which can precisely define the uniqueness among subpopulations of cells using open chromatin datasets. Using this tool, we simultaneously evaluated chromatin accessibility and gene expression datasets to estimate cell types and their respective proportions in a mixture of samples. In comparison to other known deconvolution methods, we observed the lowest average root-mean-square error (RMSE = 0.042) and the highest average correlation coefficient (*r* = 0.919) between the prediction and “true” proportion. As a proof-of-concept, we also tested chromatin accessibility data from acute myeloid leukemia (AML) and successfully obtained unique cell types associated with AML progression. Furthermore, we showed that chromatin accessibility represents more essential characteristics in the identification of cell types than gene expression. Taken together, DeconPeaker as a powerful tool has the potential to combine different datasets (primarily, chromatin accessibility and gene expression) and define different cell types in mixtures. The Python package of DeconPeaker is now available at https://github.com/lihuamei/DeconPeaker.

## Introduction

Human diseases are multifactorial and complex processes in which genetic–epigenetic components are significantly involved. To date, several key biological pathways regulating cellular functions have been defined; however, the knowledge about the behavior of individual cells is still very limited. Furthermore, the diversity among intracellular and intercellular interactions creates a significant challenge toward understanding of this multicellular network. To mention, the lack of defined gene signature and biological characteristics of bulk tissues from the histological district subtypes lead to the suboptimal–mediocre results in human diseases (Amit et al., 2020).

Several disease association studies have suggested the cell type composition as a confounding factor (Newman et al., 2015). For instance, at various stages of acute myeloid leukemia (AML), dynamic changes in cell composition from hematopoietic stem cells (HSCs) to monocytes, indicating that leukemogenesis largely mirrors the process of normal myelopoiesis (Corces et al., 2016). Likewise, the cell types in tumor microenvironment (TME) reflect both cancer subtype and the immune response (Hutter and Zenklusen, 2018). Embryogenesis, morphogenesis, cell differentiation, and growth are also directly associated with the changes in cell type composition (Hunt et al., 2019). In single-cell sequencing analysis, cell identity is mainly tagged/labeled with cell type-specific surface markers (proteins); however, the difficulties arise when heterogeneous mixture of cells also contains the unknown cell type. Furthermore, in publically available databases such as The Cancer Genome Atlas (TCGA), thousands of samples have been determined. However, these samples were generated as a mixture from bulk sequencing. Therefore, resolving cell types and compositions from these available samples will facilitate our understanding of biological mechanisms. Thus, adequate methods are needed to identify the correct cell types and compositions from a mixture.

To gain better statistical insight into the composition of the cell types in a sample mixture, many methods (also known as cell type deconvolution) have previously been developed. Most of these approaches use gene expression data by focusing on estimating the proportions and/or pure expression states, which can be divided into two subclasses, “partial” and “complete” (Gaujoux and Seoighe, 2012; Chikina et al., 2015). The former requires either cell type-specific signatures or their relative proportions (Abbas et al., 2009; Erkkilä et al., 2010; Newman et al., 2015; Hunt et al., 2019), while the latter estimates the relative cell fractions and simultaneously disentangle their expression profiles directly from mixtures (Repsilber et al., 2010; Zhong et al., 2013). In addition, DNA methylation signal is also used to predict cellular components. Houseman et al. proposed a method based on linking two regression models for the prediction of blood cell-type components (Houseman et al., 2012). Jaffe and Irizarry further reported an adaptation of the Houseman method for application to Illumina M450 array data (Aryee et al., 2014). Salas et al. proposed an optimized library for whole-blood deconvolution (Salas et al., 2018). Likewise, Chakravarthy et al. suggested about DNA methylation-based approach for the deconvolution of Pan-cancer datasets (Newman et al., 2015; Chakravarthy et al., 2018). In recent years, it has been found that chromatin accessibility at the regions distant from transcription start sites (TSSs; such as enhancers) is more predictive of cell identity than gene expression itself (Song et al., 2011; Hnisz et al., 2013). In this regard, Corces et al. demonstrated that chromatin accessibility was more cell type-specific and could capture cell identity better than mRNA expression (Corces et al., 2016). Likewise, Zamanighomi et al. suggested that cell type-specific peaks enriched the transcription factor motifs and were based on the assay for transposase-accessible chromatin by sequencing (ATAC-Seq) specific to each cell subpopulation (Zamanighomi et al., 2018).

Considering these factors, we generated DeconPeaker, a partial deconvolution method that resolves relative proportions of different cell types in the peak intensity profiles (chromatin accessibility) from the measurement of mixture samples. Compared to other known deconvolution methods, DeconPeaker is reliable and applicable to both chromatin accessibility as well as gene expression data from mixtures.

## Materials and Methods

DeconPeaker estimates relative proportions of each cell type from bulk ATAC-Seq data. The model can be simply described as *m = B × f*, where *m* is the measurement for the mixtures, *B* represents the signature matrix, and *f* denotes a vector of unknown proportions that needs to be estimated (Newman et al., 2015).

### Pre-processing for ATAC-Seq Data

ATAC-Seq datasets were processed by Kundaje’s pipeline with default parameters^1^. Briefly, the pipeline has two steps: (1) to align pair-end reads to the hg19 genome and remove duplicate reads; (2) to call narrow peaks with MACS2 (Zhang et al., 2008) for each unique cell type. Only peak and BAM files of all samples were retained.

For reference samples, we first filtered out weak peaks with −lgq ≤ 2 (defined by MACS2) to avoid false positives as previously described (Jalili et al., 2015). The peaks that overlapped with the blacklisted regions^2^ were also discarded. After generating the list of non-redundant peaks for all reference samples as previously described (Corces et al., 2016), a tool featureCounts (Liao et al., 2013) was used to calculate the fragment counts (paired reads counts) for each sample across all non-redundant peaks, resulting in a count matrix, in which rows represented peaks, and columns indicated the reference samples.

### Cell Type-Specific Peaks and Signature Matrix

To avoid batch effects, we performed quantile normalization on the count matrix across all reference samples and excluded peaks below the median value of globally normalized intensities.

#### Cell Type-Specific Peaks (CTSPs)

A statistically reliable and significant CTSP represents an open chromatin region that tends to be more pronounced in one cell type than others. Recently, Zamanighomi et al. used a Poisson regression model combined with hypothesis testing to call CTSPs in single-cell ATAC-seq (scATAC-Seq) data (Zamanighomi et al., 2018), which was difficult to converge during parameter estimation. Here, we employed the strategy of scABC (Zamanighomi et al., 2018) and gave a relatively simple and fast hypothesis-testing framework to identify CTSPs. The detailed mathematical processes can be found in Supplementary Material S1. Finally, the intensity of each CTSP was represented with the average of the peaks in each cell type’s samples.

#### Signature Matrix

**FIGURE 1:**
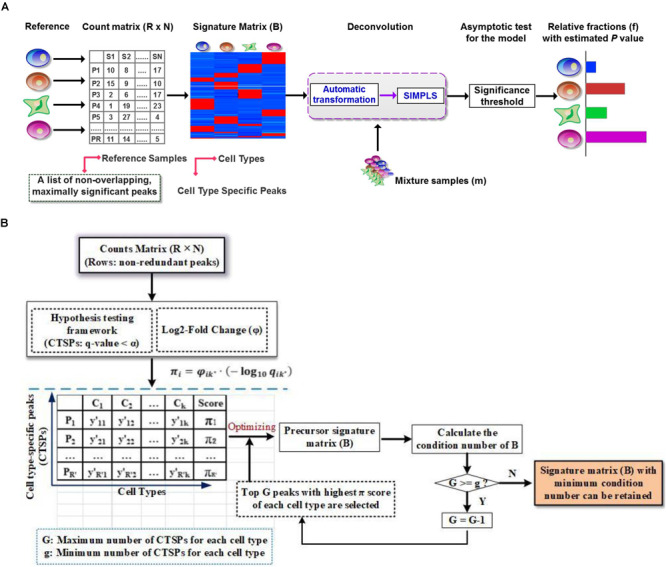
Illustration of DeconPeaker in identifying cell types using chromatin accessibility data. **(A)** Schematic of DeconPeaker. **(B)** DeconPeaker’s strategy to derive signature matrices.

This is to undermine *B*, which represents the signature matrix, as mentioned above. In a typical human ATCA-Seq, thousands of CTSPs can introduce noise, which can be avoided by shrinking the total number of CTSPs simultaneously, while considering the stability of the linear system to avoid extreme sensitivity to small fluctuations (Abbas et al., 2009; Newman et al., 2015). As previously described (Newman et al., 2015), signature matrices can be made more robust for deconvolution by minimizing condition number that is an inherent matrix property. Here, we first calculated the significance score π-value (defined in Supplementary Material S1) of each peak and then employed the optimizing strategy of CIBERSORT (Newman et al., 2015) to derive the signature matrix (Figure 1B). Briefly, we pre-set the minimum (g) and maximum (G) number of CTSPs for each cell type in *B*. For each cell type, CTSPs were ranked by π-value, and the top G CTSPs were selected into *B*. To ensure signature matrix stability, we iterated *B* across all cell types from g to G, and the signature matrix with the minimum condition number was retained. For each iteration, we performed the z-score transformation on *B.*

### Data Transformation

Owing to the complexity and diversity of the feeding data [i.e., signature matrices (*B*) and mixtures (*m*)], the accuracy and reliability of the deconvolution are difficult to guarantee. Therefore, we employed an automatic transformation method to enhance the adaptability of DeconPeaker, primarily based on the optimal parameter λ, estimated by the Box–Cox algorithm (Box and Cox, 1964). We only considered three transformation scenarios, log2-transformation (λ = 0), square-root transformation (λ = 0.5), and no transformation (λ = 1) (Osborne, 2010).

### SIMPLS for Deconvolution

In DeconPeaker, SIMPLS (De Jong, 1993) was employed to identify cell types by estimating relative subsets of chromatin accessibility. SIMPLS is a variant of partial least squares (PLS) for multiple response variables, which estimates the regression coefficients by linking signature matrices (*B*) and the measurement of the mixture (*m*), which was conducted using the function “mvr” in the R packages “pls” (Mevik et al., 2011). To obtain the estimated cell type proportion (*f*), negative regression coefficients were set to 0, and the remaining coefficients were normalized to sum to 1.

### Model Evaluation

Root-mean-square error (RMSE) and Pearson correlation coefficient (PCC) between the prediction (*f*_p_) and the known composition of cell type (*f*_t_) were calculated to evaluate the model, which were commonly used to measure differences between the ground truth and estimate. In addition, we employed an asymptotic test for the Wasserstein distance between observer *m* and prediction $\hat{\text{m}}$ to produce a *P*-value of the deconvolution using Monte Carlo sampling. Wasserstein metric reflects the degree of similarity between two distributions, in which smaller differences and smaller assigned *P*-values indicated greater significance of the model. The details of estimating *P*-value are described in Supplementary Material S1.

### Synthetic Dataset

We simulated 195 synthetic mixture samples with cell type-known ATAC-Seq data by sampling the cell type fractions using Dirichlet distribution from primary blood cells (GSE74912) with SAMTOOLS (Li et al., 2009). Cell types with different variability (1–13 types) were covered in the synthetic samples, and each synthesized sample consists of 2 million paired-end reads.

To further validate the performance of DeconPeaker using chromatin accessibility data, we retrieved a dataset of transposase-accessible chromatin profiles for 695 individual mouse cardiac progenitor cells (PRJEB23303) covering E7.5 to E9.5 of five cell types (Jia et al., 2018). From this scATAC-Seq dataset, we randomly selected 50% of cells in each cell type without replacement to construct the reference samples, and the remaining were used to synthesize 100 test mixture samples using replacement sampling. Each synthetic sample consisted of 3,000 cells. In each test sample, the proportion of each cell type was estimated with the number of five cell types in the sample.

### Data Availability

The study utilized 11 datasets from three different platforms (ATAC-Seq, RNA-Seq, and Microarray), as demonstrated in Supplementary Table S1. For the evaluation of DeconPeaker, ATAC-Seq datasets were downloaded from Gene Expression Omnibus (GEO) via accession number GSE74912 (Corces et al., 2016) and from https://github.com/loosolab/cardiac-progenitors (Jia et al., 2018). The former contains 79 normal samples and 42 AML samples (as mixture samples). Data of the normal samples, which contained 13 cell types, were used as a reference to derive the signature matrix and to generate simulated datasets with variable numbers and proportions. The latter (PRJEB23303) contained 695 cells, covering five cell types that were characterized as mouse cardiac progenitor cells from E7.5 to E9.5 using single-cell transposase-accessible chromatin profiling (scATAC-Seq). In addition to the RNA-Seq dataset (GSE74246), the mRNA expression data that matched the dataset of GSE74912 (ATAC-Seq data) was used to test the model. For comparative validation, eight benchmarking datasets were retrieved from the previously described source link^3^ (Hunt et al., 2019). Among these eight datasets, two [PRJEB8231 (Parsons et al., 2015) and GSE64098 (Ruijie et al., 2015)] were RNA-Seq data, while the other six (GSE29832 (Gong et al., 2011), GSE19830 (Shen-Orr et al., 2010), GSE11058 (Abbas et al., 2009), GSE5350 (Leming et al., 2006), GSE19380 (Kuhn et al., 2011), and GSE65133 (Newman et al., 2015)] were microarray data. Each dataset contains reference and mixture samples with known mixing proportions.

## Results

DeconPeaker predicts the cell type composition using SIMPLS (De Jong, 1993) on the basis of a signature matrix that represents cell type-specific peaks (open chromatin regions). The data processing in this tool requires three main steps (as shown in Figure 1A): (1) identification of a list of non-overlapping cell type-specific peaks (CTSPs) with the reference samples by a hypothesis test framework, then a construction of a signature matrix by minimizing the condition number (workflow shown in Figure 1B, see section “Materials and Methods”); (2) deconvolution of the mixtures with the signature matrix using SIMPLS; and (3) evaluation of the deconvolution using asymptotic test for consistency of the distributions between observations and predictions (see section “Materials and Methods”). Using these parameters, DeconPeaker can optimize chromatin accessibility data as well as cell type-specific gene expression (mRNA expression levels). The addition of SIMPLS in this tool provided a uniqueness to the deconvolution of mixed cell samples.

Notably, optimizing the number of CTSPs ensures that the signature matrix is stable and robust. In addition, the model automatically transforms the feeding data, including signature matrices, and mixtures before SIMPLS (De Jong, 1993) are applied (see section “Materials and Methods”), so as to make them fit a normal distribution as possible. As such, this strategy can enhance the adaptability and accuracy of DeconPeaker for different datasets.

### Performance Evaluation of Synthetic Mixtures

**FIGURE 2:**
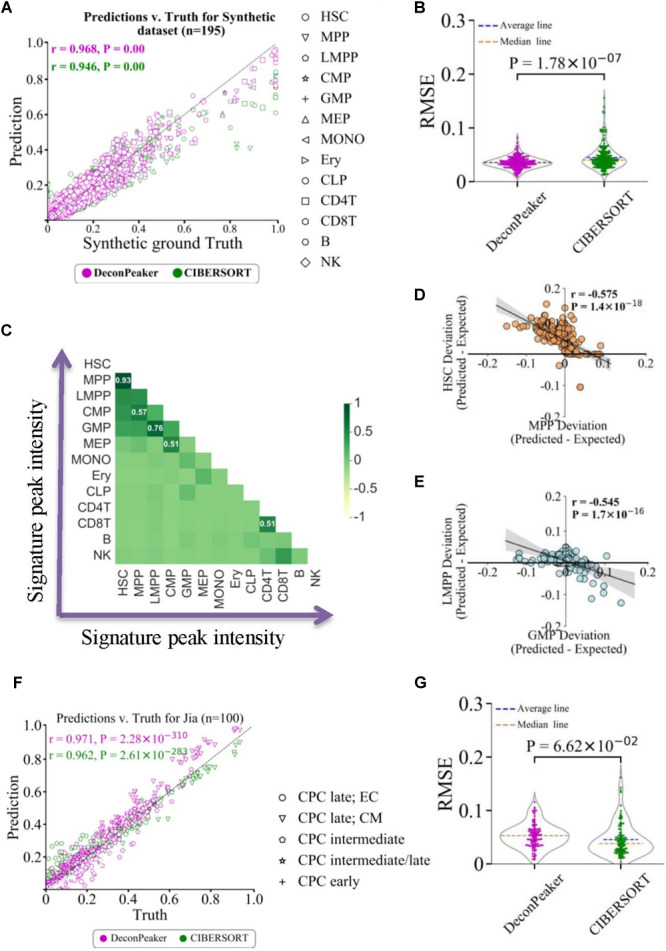
Comparison between DeconPeaker and CIBERSORT on synthetic mixtures. **(A)** Scatter plot indicating true proportions against predicted proportions by DeconPeaker and CIBERSORT in synthetic mixtures. Each point represents a specific cell type in the sample. Pearson correlation coefficient (r) was calculated between the true proportions and predictions. **(B)** Violin plot of root-mean-square error (RMSE) distribution for DeconPeaker and CIBERSORT. Each point represents a synthetic mixture sample. **(C)** Heat map showing pairwise correlation coefficients for signature peak intensities of cell types. **(D,E)** Correlation of deviations in expected subtracted prediction between HSC and MPP. Each point represents a specific cell type in the sample. Pearson correlation coefficient (r) was calculated between paired cell type deviations. **(F)** Scatter plot indicating true proportions against predicted proportions by DeconPeaker and CIBERSORT in synthetic mixtures generated using the scATAC dataset (retrieved from Jia et al.) (Jia et al., 2018). **(G)** Violin plot of RMSE distribution for DeconPeaker and CIBERSORT. Each point represents a synthetic mixture sample.

Given *n* samples for a cell type in the reference data (GSE74912) (Corces et al., 2016), *n*−1 reference samples were used to derive a signature matrix, and the remaining reference sample was used to synthesize 195 mixture samples (see “Materials and Methods,” as shown in Supplementary Figure S1). RMSE and PCC were introduced to measure the consistency between the ground truth and estimated fractions. Compared to CIBERSORT (Newman et al., 2015), DeconPeaker showed higher PCC (Figure 2A) and lower average RMSEs on the synthetic mixtures (Figure 2B). Furthermore, when decomposing the deconvolution at a single cell-type level, we found that DeconPeaker’s PCCs were above 0.95 and higher than that of CIBERSORT in predictions for each cell type (Supplementary Figures S3A–C), indicating that DeconPeaker had better deconvolution performance on synthetic mixtures of chromatin accessibility.

Additionally, the signature peak intensities showed a strong positive correlation between the pairs HSC and multipotent progenitor (MPP), granulocyte–monocyte progenitor (GMP), and lymphoid–primed multipotent progenitor (LMPP) (Figure 2C), which lead us to speculate whether this positive correlation influences the deconvolution due to the multicollinearity between the two cell types. To test multicollinearity, we first calculated the differences between the expected (truth) and predicted multicollinearity for each cell type. We also fitted the difference between the two cell types, and found a strong anti-correlation between pairs HSC–MPP and GMP–LMPP (Figures 2D,E), suggesting that multicollinearity could affect the accuracy of deconvolution if the two cell types coexist in the sample. We also compared the PCCs of the signature peak intensities between cell types with the schematic (cell lineage) of the human hematopoietic hierarchy for 13 primary blood cells types (Corces et al., 2016). The result showed that cell type pairs with strong PCC have narrow lineage distances, indicating the distance between cell types in the lineage as an important cause of multicollinearity source of potential interference in the deconvolution.

The performance of DeconPeaker was also validated on single-cell ATAC-Seq datasets (PRJEB23303). This dataset contained ATAC-Seq data for 695 mouse single cardiac progenitor cells covering E7.5 to E9.5 of five cell types (Jia et al., 2018) (see “Materials and Methods”). On 100 synthetic mixtures, DeconPeaker displayed a higher correlation coefficient (PCC = 0.97) between the truth and the predicted than CIBERSORT (Figure 2F). For the average RMSEs, the two models showed a comparable result (Figure 2G), indicating the potential of DeconPeaker in resolving the single-cell data.

### Evaluation on Experimental Data Sets

Since lack of ATAC-Seq data of cell type proportion-known mixture samples, the evaluation of our tool is based on eight known gene expression benchmarking datasets (two RNA-Seq and six microarray data), which have been widely used to test deconvolution algorithms (Supplementary Table S1). Although, DeconPeaker is modeled specifically for chromatin accessibility, except for constructing the count matrix, it shares many features similar to the other partial deconvolution algorithms (Figure 1A). This allowed us to evaluate its performance on these benchmarking datasets, even though they are not the peaks of chromatin accessibility.

**FIGURE 3:**
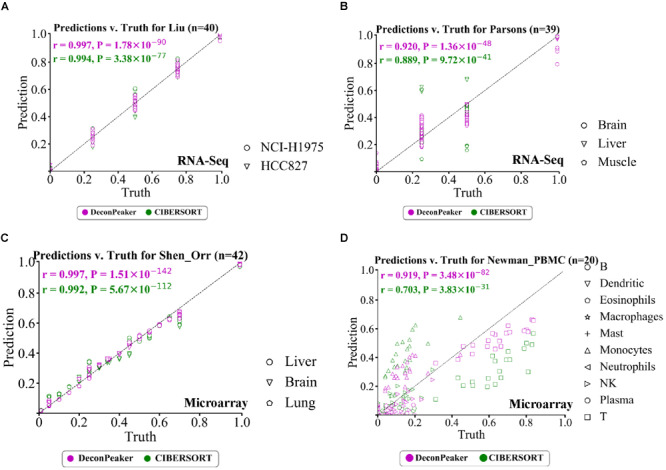
Performance of DeconPeaker and CIBERSORT on four test datasets. **(A–D)** Scatter plot indicating true proportions against predicted proportions by DeconPeaker and CIBERSORT in synthetic mixtures. Each point represents a specific cell type in a sample. Pearson correlation coefficient (r) was calculated between the true proportions and the prediction. Subplots **(A)**, **(B)**, **(C)**, and **(D)** are for Liu’s **(A)**, Parsons’ **(B)**, Shen-Orr’s **(C)**, and Newman–PBMC **(D)** datasets, respectively.

We used DeconPeaker and CIBERSORT to derive signature matrices with the reference samples of each benchmarking dataset to predict the cell type proportions in each mixed sample. In our analysis for RNA-Seq, for Liu’s data, both DeconPeaker and CIBERSORT showed good performance (Figure 3A), while in case of Parsons’ data, CIBERSORT performed slightly better (Figure 3B). Furthermore, the performances between these two tools were equally comparable for the mixture of Shen-Orr’s data, which consisted of microarray data of rat liver, brain, and lung (Figure 3C). We also tested using Newman–PBMC data, which has a very complex cell type composition and poses a huge challenge for deconvolution methods. Interestingly, DeconPeaker showed high PCC and performed better than CIBERSORT (Figure 3D). Moreover, DeconPeaker consistently showed higher PCCs than CIBERSORT in other four microarray datasets used in this study (Supplementary Figures S4A–D). To further test whether the performance of DeconPeaker is significantly better than CIBERSORT, we used a non-parametric test (Wilcoxon test) between every two groups. The results showed that the prediction by DeconPeaker significantly outperforms that by CIBERSORT on Synthetic’s, Shi’s and Shen-Orr’s, but is slightly lower on Jia’s and Kuhn’s. On the remaining datasets, the predictions of the two methods did not show any significant difference (Supplementary Figure S5). All of these clearly demonstrated the reliable performance of DeconPeaker on the benchmarking datasets in context to cross-platform adaptability.

Notably, from the RMSE perspective, the predictions on datasets of Parsons’, Shi’s, and Newman’s showed higher deviations from true proportions than on others (Supplementary Figure S5). Several factors that may make these datasets difficult to resolve: the first factor could possibly be the number of cell types in the mixed sample, while the second factor could well be the existence of two or more cell types that may be very similar, such as HSC–MPP (Figures 2C–E). An additional third factor can be considered as a batch bias of the reference profiles for the cell types.

### Comparison With Other Deconvolution Algorithms

We compared DeconPeaker against nine other deconvolution models, four of which were accessed through the CellMix R package (Gaujoux and Seoighe, 2013), including ls-fit (Abbas et al., 2009), qprog (Gong et al., 2011), DSA (Zhong et al., 2013), and deconf (Repsilber et al., 2010). The remaining algorithms, EPIC (Racle et al., 2017), PERT (Qiao et al., 2012), dtangle (Hunt et al., 2019), DeconRNASeq (Gong and Szustakowski, 2013), and CIBERSORT (Newman et al., 2015), were retrieved from the links provided in the corresponding literature. All of these models require signature genes or peaks (chromatin accessibility). However, DSA and deconf are complete deconvolution methods that only require signature genes and do not explicitly require reference data. To better evaluate and compare the performance of these methods, we have considered two scenarios. One is the comparisons of the methods based on different signature matrices. Some deconvolution methods have plugins for directly inferring signature matrices, such as CIBERSORT and dtangle, where the signature matrix is the major determinant of prediction accuracy. The second is to compare the performance of the methods using unified signature matrices.

For the first scenario mentioned above, the acquisition of signatures corresponding to different methods includes the following aspects. For DeconPeaker, CIBERSORT, and dtangle, the signature matrices were derived by their own specific strategy, while in the case of other models, the signature peaks (or genes) or signature matrices were provided by CIBERSORT. Since, some algorithms are preferred by certain platforms, such as CIBERSORT (to microarray), DeconRNASeq (to RNA-Seq), and dtangle (to both). If the deconvolution algorithms are applied to cross-platform data, their prior assumptions in the models may be destroyed. In this comparison, we have considered different configurations of the algorithms to reduce the impacts of cross-platforms on the models, and the details are provided in Supplementary Table S2.

**FIGURE 4:**
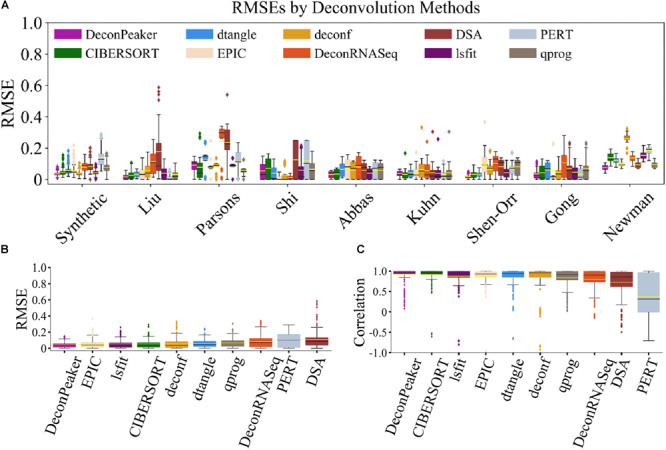
Comparison of DeconPeaker to other algorithms. **(A)** Box plots showing RMSE distribution of the predictions by algorithms on each benchmarking dataset. The yellow line in each box plot represents the average of RMSEs, while the black line is the median value. Each outlier point represents a specific mixture sample. **(B)** Side-by-side box plots indicating RMSEs in all benchmarking datasets. The yellow line in each box plot represents the average of RMSEs, while the black line is the median value. From DeconPeaker to DSA, they are sorted in ascending order based on average RMSE. Each outlier point represents a specific mixture sample. **(C)** Side-by-side box plots indicating correlations in all benchmarking datasets. The yellow line in each box plot represents the average of PCCs, while the black line is the median value. From DeconPeaker to PERT, they are sorted in descending order based on average PCC. Each outlier point is a specific sample.

We assessed the capacity of these algorithms by RMSE and PCC on the nine benchmarking datasets (include one synthetic dataset) and found that DeconPeaker showed the lowest average RMSEs on the Shen-Orr’s and Newman’s data. Although DeconPeaker performed slightly low on Shi’s and Parsons’ data, it still performed quite well (RMSE_Shi’s_ = 0.054, RMSE_Parson’s_ = 0.091) (Figure 4A), indicating good robustness. To make comparisons between the algorithms more intuitively, we combined the deconvolution of each algorithm on the benchmarking datasets, showing that DeconPeaker exhibits the lowest average RMSEs (Figure 4B) and the highest average PCCs (Figure 4C). For the second scenario, we uniformly used the signature matrices identified by DeconPeaker to evaluate the accuracy and robustness of the methods. The results showed that DeconPeaker has the second lowest average RMSEs and the highest average PCCs (Supplementary Figure S6). All of these indicate that DeconPeaker performs comparable or even better in the predictive performances. Flowchart of the analysis is shown in Supplementary Figure S2.

### Performance Evaluation of Signature Matrices

**FIGURE 5:**
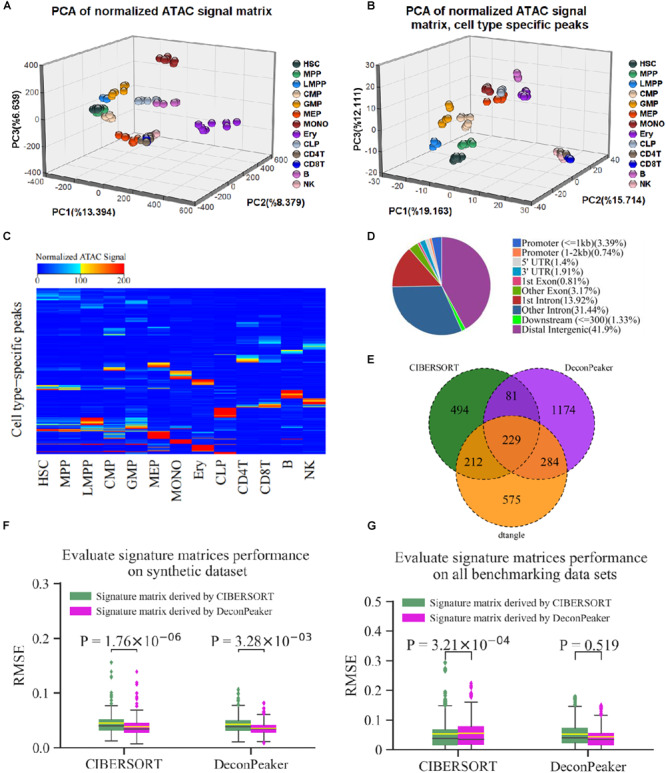
Peaks of signature matrix derived by DeconPeaker representing essential characteristics of cell identity. **(A,B)** Principal component analysis (PCA) for all non-redundant peaks **(A)** and the peaks of signature matrix **(B)**, respectively. **(C)** Heat map of signature matrix; the upper bound of the color bar is 200, and all peaks with intensity greater than 200 are colored red. **(D)** Venn plot indicating the common signature peaks identified by DeconPeaker, CIBERSORT, and dtangle. **(E)** Distribution of signature peaks by DeconPeaker using chromatin accessibility data (GSE74912). **(F)** Box plots indicating RMSE distribution of deconvolution results by DeconPeaker and CIBERSORT in synthetic mixtures. Significance was assessed by one-way ANOVA. The yellow line in each box plot represents the average of RMSEs, while the black line is the median value. Each outlier point represents a specific mixture sample. **(G)** Box plots indicating RMSE distribution of deconvolution results by DeconPeaker and CIBERSORT on the benchmarking datasets, respectively. Significance was assessed by one-way ANOVA. The yellow line in each box plot represents the average of RMSEs, while the black line is the median value. Each outlier point represents a specific mixture sample.

To evaluate the effect of signature matrices derived by DeconPeaker, the signature matrix containing 1,768 peaks from GSE74912 (Corces et al., 2016) was used. We first employed ChIPSeeker (Yu et al., 2015) to annotate the signature peaks and found that most of the peaks were located in the introns and distal intergenic regions, of which only 4.13% of the peaks were at the promoters (Figure 5D and Supplementary Table S3). This peak distribution confirms that distal element accessibility is highly cell type specific (Corces et al., 2016). Furthermore, we used principal component analysis (PCA) to visualize the distribution of individual samples of different cell types for all the peaks and signature peaks (Figures 5A,B). The clustering based on all peaks does not clearly separate cell types in the first three principal components. In contrast, the use of the peaks in the signature matrix led to a clear separation of cell type. In addition, the heat map of the signature matrix intensity intuitively showed differential peaks across the cell types (Figure 5C). Collectively, these results verify that the signature matrices derived by our model are reasonable.

It is well established that the signature matrices are the key to deconvolution. The comparison between matrices can reflect the performance of any model from different aspects. Moreover, the different strategies have been used to call the signature matrices. For instance, CIBERSORT uses a two-sided unequal variance *t*-test by minimizing condition number to derive signature matrices (Newman et al., 2015). dtangle uses the “Ratio” method, which selects and ranks markers according to the ratio of the mean expression of each gene in each cell type along with the mean of the gene in all other cell types (Hunt et al., 2019). In this study, we used Jaccard similarity coefficient (JSC) to compare the signature matrices derived by CIBERSORT, dtangle, and DeconPeaker. JSC is a measure of similarity between the two sets and defined as the number of the intersection divided by the number of the union. The results showed that the JSC between DeconPeaker and CIBERSORT is 0.125, between DeconPeaker and dtangle is 0.201, and between CIBERSORT and dtangle is 0.235 (Figure 5E). The low JSCs suggest that the signature peaks derived by different algorithms vary widely.

Furthermore, we exchanged the signature matrices derived by DeconPeaker and CIBERSORT, and compared the deconvolution on the synthetic mixtures. The signature matrix identified by DeconPeaker had lower average RMSEs, indicating that the signature matrices identified by DeconPeaker can improve the accuracy of deconvolution (Figure 5F). In addition, the deconvolutions by CIBERSORT using an external and the self-identified signature matrices showed different average RMSEs by one-way ANOVA, while DeconPeaker was found to be relatively stable (Figure 5F). To further explore this issue, we used all benchmarking datasets to test. It was found that DeconPeaker is stable against the external signature matrices, while CIBERSORT is sensitive (Figure 5G). When using the signature matrices identified by DeconPeaker, the RMSEs of both algorithms were smaller in median RMSEs and had more compact distribution, although the average RMSEs were slightly higher in CIBERSORT (Figure 5G). Taken together, the signature matrices derived by DeconPeaker showed good performance in deconvolution. Furthermore, DeconPeaker’s deconvolution based on the external signature matrix maintained good stability and accuracy.

### Impact of Data Transformation

**FIGURE 6:**
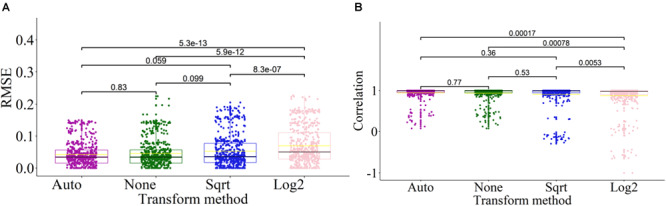
Effects of transformation methods on deconvolution. **(A)** Side-by-side box plots indicating RMSE distribution of deconvolution results by transformation methods on all benchmarking datasets. Each outlier point represents a specific mixture sample. Significance was assessed by Wilcoxon test. **(B)** Side-by-side box plots indicating distribution of correlations of deconvolution results by transformation methods on all benchmarking datasets. Each outlier point represents a specific mixture sample. Significance was assessed by Wilcoxon test.

To enhance the adaptability of DeconPeaker to different kinds of datasets (gene expression or open chromatin), an automatic data transformation method was introduced, and the deconvolution performance was evaluated. Here, we only applied “None” (without transform), “Auto” (Automatic transform), “Log2” (log2-transform), and “Sqrt” (square-root transform) transformations on the signature matrix and the mixtures of each benchmarking data. The results indicated that transformation “Auto” has the lowest average RMSEs, the highest average PCCs, and the most compact distribution (Figures 6A,B), indicating that the automatic transformation method could improve the adaptability and deconvolution performance for different kinds of datasets. To test if there is significant difference between the transform methods, we introduced Wilcoxon test between every two groups. The results showed that there were significant differences between “Log2” and the others, and no significant differences among “Auto,” “Sqrt,” and “None” (Figures 6A,B). Hence, in most cases, the peak profile or gene expression deconvolution should be done in linear space rather than log-transformed space. However, the model that combines multiple transformation strategies has better data adaptability for deconvolution. A flowchart of this analysis is shown in Supplementary Figure S2.

### Deconvolution of Cell Composition for AML Dataset

We used a dataset of HSCs from AML (Corces et al., 2016). This dataset contained 32 unique mixture samples (replicates were merged) on ATAC-Seq and RNA-Seq, primarily covering three distinct stages of AML development, namely, preleukemic HSCs (pHSCs), leukemia stem cells (LSCs), and leukemic blast cells (Blasts). According to the literature (Corces et al., 2016), cell type compositions were mostly HSCs and MPPs in pHSCs, and GMPs and LMPPs in LSCs. However, the Blasts analysis showed a wider distribution, namely, less differentiated blasts associate with GMP cells, and more differentiated blasts associate with monocytes (MONOs) (Corces et al., 2016).

**FIGURE 7:**
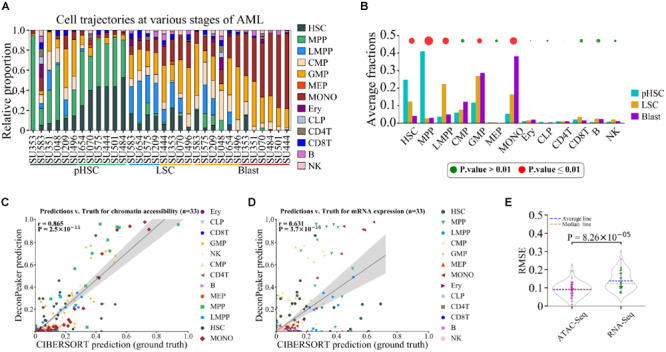
Deconvolution of cell composition using AML chromatin accessibility data. **(A)** Deconvolution showing the predicted contribution of various normal cell types to the chromatin accessibility landscape of AML. pHSC, LSC, and Blast represent three distinct stages in AML development. **(B)** Average proportions of predicted cell types in each stage of AML. Points above the bars indicate statistical significant changes for each cell type in the three stages. Size of the point is equal to -lg*p*, where *p* was assessed by one-way ANOVA. **(C)** Correlation between AML predictions by DeconPeaker and CIBERSORT used in Corces et al. (Corces et al., 2016) (served as ground truth) on chromatin accessibility data. Each point represents a specific cell type in a sample. Pearson correlation coefficient (r) was calculated between the predictions of CIBERSORT and DeconPeaker. **(D)** Correlation between AML predictions by DeconPeaker and CIBERSORT used in Corces et al. (served as ground truth) on mRNA expression data. Each point represents a specific cell type in a sample. Pearson correlation coefficient (r) was calculated between the predictions of CIBERSORT and DeconPeaker. **(E)** Side-by-side box plots indicating RMSEs in ATAC-Seq and RNA-Seq for AML. Each point represents a specific platform AML sample. Significance was assessed by one-way ANOVA.

For the ATAC-Seq data (GSE74912), we built the signature matrix (1,768 peaks) based on 77 normal samples, covering 13 primary blood cell types (workflow shown in Supplementary Figure S1), and used this signature matrix to deconvolute the mixtures of AML (Figures 7A,B). HSC and MPP were found as the major cell type components of the pHSC stage, the proportions of LMPP and GMP showed a significant abundance in the LSCs stage, and GMP and MONO dominated the Blast stage, which is consistent with the previous report (Corces et al., 2016). In addition, we observed an increase in the MONO phase in the three stages, especially from the LSC stage to the Blast stage. Notably, GMP showed no significant change between LSC and Blast stages.

For RNA-Seq data (GSE74246), we built the signature matrix (1,245 genes) with 49 normal transcriptomes to deconvolute the mixed transcriptomes of AML (Supplementary Figure S1). The results showed that HSC and MPP dominated the pHSC stage (Supplementary Figures S7A,B), which is consistent with the finding based on ATAC-Seq data (Figures 7A,B). The proportion of LMPP was the highest in the LSC stage, while MONO dominated in the Blast stage. According to literature (Corces et al., 2016) and the deconvolution results for ATAC-Seq data (Figures 7A,B), a high proportion of GMP was found in both LSC and Blast stages, but in the deconvolution using RNA-Seq data, GMP did not show a dominating proportion, suggesting that gene expression is not the most essential feature of cell identity.

To further evaluate the capacity of chromatin accessibility data and gene expression data to identify cell types, we visualized the distribution of individual samples of different cell types with PCA, using either the signature peaks (from GSE74912) (Figure 5B) or the signature genes (from GSE74246) (Supplementary Figure S7C). In PCA plots when using cell type-specific ATAC-Seq peaks, samples of the same cell type were better clustered, while the ones of different cell types were better separated in comparison to the results with the signature genes, such as for cell types LMPP, GMP, and CMP (Figure 5B and Supplementary Figure S7C), suggesting that the chromatin accessibility is more specific in classifying cell types. Furthermore, to compare the performance of deconvolution of the mixed samples on the two signature matrices, the literature results were used as a standard cell type proportion (Corces et al., 2016) and were further compared to the cell type proportions predicted either based on ATAC-Seq data or on RNA-Seq data by calculating the correlation (PCC) between them. The results showed that the cell type proportion based on ATAC-Seq data exhibited a higher PCC and lower average RMSEs with the standard cell type proportion compared to that based on RNA-Seq data (Figures 7C–E). This further confirmed that chromatin accessibility provides more information about the cell identity than gene expression. In addition, we identified the overlapping genes between the signature genes and the genes associated with the signature ATAC-Seq peaks. According to the annotation (Supplementary Table S3), the 1,768 signature ATAC-Seq peaks associate with the 870 genes. The number of the expression signature genes is 1,245 (GSE74246). Importantly, we found that only 112 genes (∼6.3% to peak number) were common between both (Supplementary Figure S7D). This small fraction (6.3%) of overlap indicates that most of the ATAC signature peaks are not at the regulatory sites of the signature genes. In other words, a chromatin accessibility region (one peak) probably corresponds to multiple genes in the regulation, not merely in a one-to-one manner, especially in 3D organization of genome. This can also be evident from the fact that gene expression is consequence of a complex regulatory process. For a refine and unique cell type or cell state, several factors (even external stimuli) often play a significant role, primarily affecting the transcription factor bindings to the DNA.

## Discussion

Gene expression deconvolution methods are ideal to define unique cell types in transcriptomes of samples with mixed cell types. Likewise, DNA methylation data is also highly cell type specific and can reveal hidden components of tissue mixtures. Studies have shown that models using DNA methylation consistently outperformed the gene expression-based methods (Houseman et al., 2012; Reinius et al., 2012; Chakravarthy et al., 2018). Notably, the accessibility of chromatin is related to the binding of the transcription factor to DNA, which also indicates cell type specificity. In this study, we generated DeconPeaker, a novel deconvolution estimator for characterizing cell type composition using chromatin accessibility mixtures, as well as for gene expression datasets (RNA-Seq and Microarray). To achieve the higher optimization with DeconPeaker, we first used multiple linear regression with intersection-union test (IUT) by minimizing the condition number to derive signature matrices and then predicted the cellular fractions of the mixed samples by SIMPLS with the derived signature matrices. In comparison to other known deconvolution methods on the benchmarking datasets across different platforms, DeconPeaker demonstrated the lowest average RMSEs and highest average PCCs between predictions and truths (Figures 4B,C). In addition, the signature matrices identified by DeconPeaker consistently showed lower average RMSEs and the highest average PCCs, indicating a greater reliability and a broader range of applications of DeconPeaker. Notably, we have proposed a novel hypothesis testing framework by minimizing condition number to identify signature matrices in DeconPeaker. Compared with CIBERSORT (Newman et al., 2015), our strategy enables us to more accurately depict cell type specificity of peaks or genes (gene expression data). The automatic transformation strategy for feeding data (signature matrices and mixtures) is unique in DeconPeaker compared to other deconvolution methods. This is due to the fact that outliers and anomalous distribution of feeding data can greatly affect the performance of the model, which is required to ensure accuracy of the deconvolution (Figures 6A,B). In most cases, the peak profile or gene expression deconvolution in linear space performs better than in log-transformed space.

To validate DeconPeaker, we analyzed marker cell types of AML at different stages based on the chromatin accessibility (ATAC-Seq, GSE74912) and mRNA expression data (GSE74246). The conclusions derived from chromatin accessibility were consistent with previous reports (Corces et al., 2016), suggesting that this tool has important applications in the interpretation and identification of biological mechanisms. We further evaluated the capacity of chromatin accessibility data and gene expression data in identifying cell types, and found that chromatin accessibility was more specific than gene expression in the classification of cell types (Supplementary Figure S7C). Moreover, deconvolution with chromatin accessibility data had higher PCC and lower average RMSEs between the predictions and the standard cell type proportion (Figures 7C–E). This indicates that chromatin accessibility represents more information about cell identity than gene expression. It is noteworthy to mention that we have found only a small number (∼6.3%) of genes that overlapped between the expression signature genes and the genes that associate ATAC-Seq signature peaks (Supplementary Figure S7D). This indicates that the cell identity in a different cellular information has a distinct feature, namely, transcription regulation layer (chromatin accessibility) and the gene expression layer. Since the cell type or cell state often associates with extracellular stimulus, the chromatin accessibility, which affects the transcription factor binding to DNA, is probably more sensitive to the cell identity.

In addition, we must also point out the possible biases when using a constant signature matrix to deconvolute samples under different disease states or sequencing platforms. Therefore, we propose a signature matrix based on purified data from multiple platforms and from both healthy and disease samples to reduce the biological and technique bias (Vallania et al., 2018).

Taken together, “DeconPeaker” is amenable to chromatin accessibility data measured with ATAC-Seq and gene expression datasets. Primarily, due to its flexible statistical approach, it will enable researchers to measure bulk biospecimens, in particular, the samples with a mixture of cell types. Notably, to enhance the performance, additional test using DeconPeaker, particularly on more ATAC-Seq datasets containing both reference and cell type proportion-known mixture samples should be conducted in the future.

## Data Availability Statement

Publicly available datasets were analyzed in this study. This data can be found at NCBI: GSE74912, GSE74246, GSE5350, GSE29832, GSE19830, GSE11058, GSE19380, GSE65133, and GSE64098. This data can be available from the ENA repository: PRJEB8231 and PRJEB23303.

## Author Contributions

HDL and XS designed the study. HML coded the algorithms. HML, HDL, and AS wrote and revised the manuscript. HML conducted the data analysis. XS, KL, and ZQ provided interpretation and discussion. All authors contributed and approved the final manuscript.

## Conflict of Interest

The authors declare that the research was conducted in the absence of any commercial or financial relationships that could be construed as a potential conflict of interest.

## Footnotes

1 https://github.com/kundajelab/atac_dnase_pipelines

2 https://www.encodeproject.org/annotations/ENCSR636HFF/

3 https://wm1693.box.com/s/np66a1wnhngafoawsiu665sjb7kye2ub

## Supplementary Material

FIGURE S1 Schematic of DeconPeaker validation on ATAC-Seq synthetic mixtures and applications on acute myeloid leukemia (AML) datasets.

FIGURE S2 Schematic of comparisons across other known deconvolution methods and evaluation of performance of different transformations.

FIGURE S3 Deconvolution with DeconPeaker providing robust accuracy of most human hematopoietic cell types in the synthetic dataset.

FIGURE S4 Performance of DeconPeaker and CIBERSORT on four microarray datasets.

FIGURE S5 Significance test of the predictions between CIBERSORT and DeconPeaker across all benchmark datasets.

FIGURE S6 Comparison of DeconPeaker to other algorithms based on the same signature peaks or genes.

FIGURE S7 Deconvolution of cell composition using AML mRNA expression data.

TABLE S1 Datasets for comparisons between deconvolution algorithms.

TABLE S2 Deconvolution configurations of compared algorithms.

TABLE S3 Annotation of signature peaks from ATAC-Seq data (GSE74912).

MATERIAL S1 Supplementary Note: Identification of cell type-specific peaks, Calculation of significance score π-value, and Asymptotic test for deconvolution.

## References

[B1] Abbas A. R. Wolslegel K. Seshasayee D. Modrusan Z. Clark H. F. (2009). Deconvolution of blood microarray data identifies cellular activation patterns in systemic lupus erythematosus. *PLoS One* 4:e6098. 10.1371/journal.pone.0006098 19568420 PMC2699551

[B2] Amit S. Heiko R. Jörg E. (2020). DNA methylation & bladder cancer: where genotype does not predict phenotype. *Curr. Genom.* 21 34–36. 10.2174/1389202921666200102163422 PMC7324896 32655296

[B3] Aryee M. J. Jaffe A. E. Corrada-Bravo H. Ladd-Acosta C. Feinberg A. P. Hansen K. D. (2014). Minfi: a flexible and comprehensive Bioconductor package for the analysis of Infinium DNA methylation microarrays. *Bioinformatics* 30 1363–1369. 10.1093/bioinformatics/btu049 24478339 PMC4016708

[B4] Box G. E. P. Cox D. R. (1964). An analysis of transformations. *J. R. Stat. Soc. Series B Stat. Methodol.* 26 211–252.

[B5] Chakravarthy A. Furness A. Joshi K. Ghorani E. Ford K. Ward M. J. (2018). Pan-cancer deconvolution of tumour composition using DNA methylation. *Nat. Commun.* 9 1–13. 10.1038/s41467-018-07155-4 30104673 PMC6089972

[B6] Chikina M. Zaslavsky E. Sealfon S. C. (2015). CellCODE: a robust latent variable approach to differential expression analysis for heterogeneous cell populations. *Bioinformatics* 31 1584–1591. 10.1093/bioinformatics/btv015 25583121 PMC4426841

[B7] Corces M. R. Buenrostro J. D. Wu B. Greenside P. G. Chan S. M. Koenig J. L. (2016). Lineage-specific, and single-cell chromatin accessibility charts human hematopoiesis and leukemia evolution. *Nat. Genet.* 48 1193–1203. 10.1038/ng.3646 27526324 PMC5042844

[B8] De Jong S. J. (1993). SIMPLS: an alternative approach to partial least squares regression. *Chemometr. Intell. Lab. Syst.* 18 251–263.

[B9] Erkkilä T. Lehmusvaara S. Ruusuvuori P. Visakorpi T. Shmulevich I. Lähdesmäki H. (2010). Probabilistic analysis of gene expression measurements from heterogeneous tissues. *Bioinformatics* 26 2571–2577. 10.1093/bioinformatics/btq406 20631160 PMC2951082

[B10] Gaujoux R. Seoighe C. (2012). Semi-supervised nonnegative matrix factorization for gene expression deconvolution: a case study. *Infect. Genet. Evol.* 12 913–921. 10.1016/j.meegid.2011.08.014 21930246

[B11] Gaujoux R. Seoighe C. (2013). CellMix: a comprehensive toolbox for gene expression deconvolution. *Bioinformatics* 29 2211–2212. 10.1093/bioinformatics/btt351 23825367

[B12] Gong T. Hartmann N. Kohane I. S. Brinkmann V. Staedtler F. Letzkus M. (2011). Optimal deconvolution of transcriptional profiling data using quadratic programming with application to complex clinical blood samples. *PLoS One* 6:e27156. 10.1371/journal.pone.0027156 22110609 PMC3217948

[B13] Gong T. Szustakowski J. D. (2013). DeconRNASeq: a statistical framework for deconvolution of heterogeneous tissue samples based on mRNA-Seq data. *Bioinformatics* 29 1083–1085. 10.1093/bioinformatics/btt090 23428642

[B14] Hnisz D. Abraham B. J. Lee T. I. Lau A. Saint-Andre V. Sigova A. A. (2013). Super-enhancers in the control of cell identity and disease. *Cell* 155 934–947. 10.1016/j.cell.2013.09.053 24119843 PMC3841062

[B15] Houseman E. A. Accomando W. P. Koestler D. C. Christensen B. C. Marsit C. J. Nelson H. H. (2012). DNA methylation arrays as surrogate measures of cell mixture distribution. *BMC Bioinformatics* 13:86. 10.1186/1471-2105-13-86 22568884 PMC3532182

[B16] Hunt G. J. Freytag S. Bahlo M. Gagnon-Bartsch J. A. (2019). Dtangle: accurate and robust cell type deconvolution. *Bioinformatics* 35 2093–2099. 10.1093/bioinformatics/bty926 30407492

[B17] Hutter C. Zenklusen J. C. (2018). The cancer genome atlas: creating lasting value beyond its data. *Cell* 173 283–285. 10.1016/j.cell.2018.03.042 29625045

[B18] Jalili V. Matteucci M. Masseroli M. Morelli M. J. (2015). Using combined evidence from replicates to evaluate ChIP-seq peaks. *Bioinformatics* 31 2761–2769. 10.1093/bioinformatics/bty119 25957351

[B19] Jia G. Preussner J. Chen X. Guenther S. Yuan X. Yekelchyk M. (2018). Single cell RNA-seq and ATAC-seq analysis of cardiac progenitor cell transition states and lineage settlement. *Nat. Commun.* 9:4877. 10.1038/s41467-018-07307-6 30451828 PMC6242939

[B20] Kuhn A. Thu D. Waldvogel H. J. Faull R. L. Luthi-Carter R. (2011). Population-specific expression analysis (PSEA) reveals molecular changes in diseased brain. *Nat. Methods* 8 945–947. 10.1038/nmeth.1710 21983921

[B21] Leming S. Reid L. H. Jones W. D. Richard S. Warrington J. A. Baker S. C. (2006). The MicroArray Quality Control (MAQC) project shows inter- and intraplatform reproducibility of gene expression measurements. *Nat. Biotechnol.* 24 1151–1161. 10.1038/nbt1239 16964229 PMC3272078

[B22] Li H. Handsaker B. Wysoker A. Fennell T. Ruan J. Homer N. (2009). The sequence alignment-map format and SAMtools. *Bioinformatics* 25 2087–2089. 10.1093/bioinformatics/btp352 19505943 PMC2723002

[B23] Liao Y. Smyth G. K. Shi W. (2013). Feature counts: an efficient general purpose program for assigning sequence reads to genomic features. *Bioinformatics* 30 923–930. 10.1093/bioinformatics/btt656 24227677

[B24] Mevik B.-H. Wehrens R. Liland K. H. J. R. p. v. (2011). *pls: Partial Least Squares and Principal Component Regression*, Vol. 2.

[B25] Newman A. M. Liu C. L. Green M. R. Gentles A. J. Feng W. Xu Y. (2015). Robust enumeration of cell subsets from tissue expression profiles. *Nat. Methods* 12 453–457. 10.1038/nmeth.3337 25822800 PMC4739640

[B26] Osborne J. W. (2010). Improving your data transformations: applying the Box-Cox transformation. *Pract. Assess Res. Eval.* 15:9.

[B27] Parsons J. Munro S. Pine P. S. Mcdaniel J. Mehaffey M. Salit M. (2015). Using mixtures of biological samples as process controls for RNA-sequencing experiments. *BMC Genomics* 16:708. 10.1186/s12864-015-1912-7 26383878 PMC4574543

[B28] Qiao W. Quon G. Csaszar E. Yu M. Morris Q. Zandstra P. W. (2012). PERT: a method for expression deconvolution of human blood samples from varied microenvironmental and developmental conditions. *PLoS Comput. Biol.* 8:e1002838. 10.1371/journal.pcbi.1002838 23284283 PMC3527275

[B29] Racle J. Jonge K. De Baumgaertner P. Speiser D. E. Gfeller D. (2017). Simultaneous enumeration of cancer and immune cell types from bulk tumor gene expression data. *eLife* 6:e26476. 10.7554/eLife.26476 29130882 PMC5718706

[B30] Reinius L. E. Acevedo N. Joerink M. Pershagen G. Dahlén S.-E. Greco D. (2012). Differential DNA methylation in purified human blood cells: implications for cell lineage and studies on disease susceptibility. *PLoS One* 7:e41361. 10.1371/journal.pone.0041361 22848472 PMC3405143

[B31] Repsilber D. Kern S. Telaar A. Walzl G. Black G. F. Selbig J. (2010). Biomarker discovery in heterogeneous tissue samples-taking the in-silico deconfounding approach. *BMC Bioinformatics* 11:27. 10.1186/1471-2105-11-27 20070912 PMC3098067

[B32] Ruijie L. Holik A. Z. Shian S. Natasha J. Kelan C. Huei San L. (2015). Why weight? Modelling sample and observational level variability improves power in RNA-seq analyses. *Nucleic Acids Res.* 43:e97. 10.1093/nar/gkv412 25925576 PMC4551905

[B33] Salas L. A. Koestler D. C. Butler R. A. Hansen H. M. Wiencke J. K. Kelsey K. T. (2018). An optimized library for reference-based deconvolution of whole-blood biospecimens assayed using the Illumina HumanMethylationEPIC BeadArray. *Genome Biol.* 19:64. 10.1186/s13059-018-1448-7 29843789 PMC5975716

[B34] Shen-Orr S. S. Tibshirani R. Khatri P. Bodian D. L. Staedtler F. Perry N. M. (2010). Cell type-specific gene expression differences in complex tissues. *Nat. Methods* 7 287–289. 10.1038/nmeth.1439 20208531 PMC3699332

[B35] Song L. Zhang Z. Grasfeder L. L. Boyle A. P. Giresi P. G. Lee B. K. (2011). Open chromatin defined by DNaseI and FAIRE identifies regulatory elements that shape cell-type identity. *Genome Res.* 21 1757–1767. 10.1101/gr.121541.111 21750106 PMC3202292

[B36] Vallania F. Tam A. Lofgren S. Schaffert S. Azad T. D. Bongen E. (2018). Leveraging heterogeneity across multiple datasets increases cell-mixture deconvolution accuracy and reduces biological and technical biases. *Nat. Commun.* 9 4735. 10.1038/s41467-018-07242-6 30413720 PMC6226523

[B37] Yu G. Wang L. G. He Q. Y. (2015). ChIPseeker: an R/Bioconductor package for ChIP peak annotation, comparison and visualization. *Bioinformatics* 31 2382–2383. 10.1093/bioinformatics/btv145 25765347

[B38] Zamanighomi M. Lin Z. Daley T. Chen X. Duren Z. Schep A. (2018). Unsupervised clustering and epigenetic classification of single cells. *Nat. Commun.* 9:2410. 10.1038/s41467-018-04629-3 29925875 PMC6010417

[B39] Zhang Y. Liu T. Meyer C. A. Eeckhoute J. Johnson D. S. Bernstein B. E. (2008). Model-based analysis of ChIP-Seq (MACS). *Genome Biol.* 9:R137. 10.1186/gb-2008-9-9-r137 PMC2592715 18798982

[B40] Zhong Y. Wan Y.-W. Pang K. Chow L. M. Liu Z. (2013). Digital sorting of complex tissues for cell type-specific gene expression profiles. *BMC Bioinformatics* 14:89. 10.1186/1471-2105-14-89 23497278 PMC3626856

