# Erratum: DeconPeaker, a Deconvolution Model to Identify Cell Types Based on Chromatin Accessibility in ATAC-Seq Data of Mixture Samples

**DOI:** 10.3389/fgene.2020.00693

**Published:** 2020-06-15

**Authors:** Frontiers Production Office

**Affiliations:** Frontiers Media SA, Lausanne, Switzerland

**Keywords:** chromatin accessibility, cell type, deconvolution, mixture samples, gene expression

Due to a typesetting error, many of the references were cited in the wrong place. In addition the citations in the legends for Figures 2F and 7C were incorrect. In the former, “Jia et al.” should be “Jia et al. (2018)”. In the latter, “Corces et al.” should be “Corces et al. (2016)”. The publisher apologizes for this mistake. The original version of this article has been updated.
