# Supplementary material for: DeconPeaker, a Deconvolution Model to Identify Cell Types Based on Chromatin Accessibility in ATAC-Seq Data of Mixture Samples
Source: Front Genet. 2020 Jun 8;11:392. doi: 10.3389/fgene.2020.00392 (PMC7269180; doi:10.3389/fgene.2020.00392)
Supplement: FIGURE S1 — Schematic of DeconPeaker validation on ATAC-Seq synthetic mixtures and applications on acute myeloid leukemia (AML) datasets. [file Data_Sheet_1.docx]

Supplementary Material

# Supplementary Note

**Identification of cell type-specific peaks**

We gave a relatively simple and fast hypothesis testing framework to identify cell type-specific peaks (CTSPs), and the details as follows.

**Model assumption:** Let K denotes the number of cell types, N denotes the number of reference samples, $x_{\mathrm{jk}}$ the cell membership for sample $j$ with $x_{\mathrm{jk}}=1$ if sample $j$ belongs to cell type $k$ and $x_{\mathrm{jk}}=0$ otherwise, $y_{\mathrm{ij}}$ the normalized intensity of peak $i$ in sample $j$. We have equation (1)

|  | $y_{\mathrm{ij}}=\beta_{0}+\sum_{k=1}^{K} x_{\mathrm{jk}}\beta_{\mathrm{ik}}$ | (1) |
| --- | --- | --- |

We fitted the model without $\beta_{0}$. Coefficient $\beta_{\mathrm{ik}}$ represents the expect effect of the members in the k-th cell type on peak $i$.

**Hypothesis testing:** The intersection-union test (IUT), a combination of $t$ tests [1], was used to find CTSPs. Supposing $\Xi_{-k}={\{1,2,\ldots, K\}}_{-k}$ represents set $\{1,2,\ldots, K\}$ except for the k-th cell type, $c_{k}$ the number of reference samples for cell type $k$, the IUT testing hypothesis of peak *i* is equation (2) :

|  | $H_{0}: \beta_{ik}\leq\beta_{ik^{'}}, for some k^{'}\in\Xi_{-k} versus H_{1}: \beta_{ik}>\beta_{ik^{'}}, for all k^{'}\in\Xi_{-k}$ | (2) |
| --- | --- | --- |

According to Hangen Koo et al. [2], for null hypothesis, the $t$ test statistic is equation (3)

|  | $t_{k^{'}}(c_{k}+c_{k^{'}}-2)=\frac{\hat{\beta_{ik}}-\hat{\beta_{ik^{'}}}}{\sqrt{\frac{{{(c}_{k}-1)(SE(\beta_{ik}))}^{2}+{{(c}_{k^{'}}-1)(SE(\beta_{ik^{'}}))}^{2}}{c_{k}+c_{k^{'}}-2}}}$ | (3) |
| --- | --- | --- |

where $SE\left( \beta_{ik} \right)=\sqrt{s_{i}^{2}{(X^{'}X)}_{kk}^{-1}}$, $X$ denotes $N\times K$ design matrix with entry $x_{jk}=1$ if sample $j$ belongs to cell type $k$ and $x_{jk}=0$ otherwise, $s_{i}^{2}$ denotes the estimated variance of the regression residuals of peak $i$. The *p*-value of $H_{0}$ for peak *i* is equation (4)

|  | $p_{i}=max\{p_{{ik}^{'}}, k^{'}\in\Xi_{-k}\}$ | (4) |
| --- | --- | --- |

Where $p_{ik^{'}}$ is the *p*-value of $H_{0k^{'}}$ for peak *i*. Finally, we used the Benjamini-Hochberg method [3] to adjust *p*-value, and those peaks with a *q*-value <$\alpha(significant level, default is 0.1)$ were considered as CTSPs.

**Calculation of significance score** $\boldsymbol{\pi}$**-value**

We took q-value (adjusted p-value) and log2-fold change (LFC) of each peak into consideration, then a significance score $\pi$-value [4] of each peak could be calculated by equation (5).

|  | $\pi_{i}=\varphi_{ik^{*}}\cdot(-\log_{10} q_{ik^{*}})$ | (5) |
| --- | --- | --- |

Where $q_{ik^{*}}$ is$min\{q_{ik}, k\in\Xi\}$, $\varphi_{ik^{*}}$ is the LFC of the $k^{*}$-th cell type on peak $i$, which compared to other cell subsets. According to eq.5, $\pi$-value is a non-negative, and the larger it is, the more significant the peak cell type-specific is. Xiao et al confirmed that $\pi$-values are more robust than *ad hoc* combination in screening differentially expressed genes on expression data [4].

**Asymptotic test for deconvolution**

We employed an asymptotic test for the consistency of distributions between observations (**m**) and predictions ($\hat{\mathbf{m}}$) by using Monte Carlo sampling to produce a *P* value of deconvolution. The details of this approach can be described as: give an observed mixture **m** and its predicted value $\hat{\mathbf{m}}$ by DeconPeaker. The distance between **m** and $\hat{\mathbf{m}}$ can be calculated by equation (6),

|  | $W\left( \mathbf{m,}\hat{\mathbf{m}} \right)=\inf_{\gamma\sim\Pi(\mathbf{m,}\hat{m})} \mathbb{E}_{(w,v )\sim\gamma}[\vert\vert w-v\vert\vert]$ | (6) |
| --- | --- | --- |

where W is the Wasserstein distance that reflects the degree of similarity between the two distributions. $\Pi(\mathbf{m,}\hat{\mathbf{m}})$ is a collection of all possible joint distributions of **m** and $\hat{\mathbf{m}}$, $(w,v )$ is a sample from the joint distribution $\gamma$. $||w-v||$ represents the distance between *w* and *v*. To derive an empirical *P* value of deconvolution, we first generated a null distribution ***R^*^***, and required two steps: 1) obtained a random mixture **m*** equal to the size of **m** from [min(**m**), max(**m**)] using uniform sampling, and 2) calculated the Wasserstein distance between **m* and** $\hat{\boldsymbol{m}^{\boldsymbol{*}}}$ and stored to ***R^*^*,** where $\hat{\boldsymbol{m}^{\boldsymbol{*}}}$is the prediction of **m^*^** based on signature matrix **B** by using DeconPeaker. The two processes were repeated ***I*** (default: 1000) iterations and then sorted ***R^*^*** by descending order. Finally, the *P* value of deconvolution could be derived by equation (7),

|  | $P\left( \boldsymbol{m,}\hat{\boldsymbol{m}} \right)=1-\frac{min(\{ind\vert if R_{ind}^{*}<W\left( \boldsymbol{m,}\hat{\boldsymbol{m}} \right), 1\leq ind\leq\boldsymbol{I} and ind\in Z^{+} \})}{\boldsymbol{I}}$ | (7) |
| --- | --- | --- |

where *ind* is the subscript of the ***R^*^***, $P\left( m, \hat{m} \right)$ is the *P* value of the deconvolution. With this method, we could derive the *P* value for each mixture sample by using DeconPeaker.

**REFERENCES**

[1] R.L. Berger, Likelihood Ratio Tests and Intersection-Union Tests, Advances in statistical decision theory and applications, 1997, pp. 225-237.

[2] H. Koo, and D.S. Hong, Class and Income Inequality in Korea. American Sociological Review 45 (1980) 610-626.

[3] Y. Benjamini, and Y.J.J.o.t.R.S.S. Hochberg, Controlling The False Discovery Rate - A Practical And Powerful Approach To Multiple Testing. 57 (1995) 289-300.

[4] Y. Xiao, T.H. Hsiao, U. Suresh, H.I. Chen, X. Wu, S.E. Wolf, and Y. Chen, A novel significance score for gene selection and ranking. Bioinformatics 30 (2014) 801-7.

# Supplementary Tables and Figures

## Supplementary Tables

**Table S1.** Data sets for comparisons between deconvolution algorithms. The column ‘Usage’ represents the usages of the data sets in this study; The column "Truth" is used to distinguish whether the proportion of each component of the mixture is known; The column “Reference” lists the number of samples in the reference data, where “internal” denotes the pure reference samples were created part and parcel with the mixture experiment, if the reference samples were collected from an external data source, then "external". Eight of these data sets were obtained directly from Hunt et al (Hunt, et al., 2018) and as benchmarking datasets.

| **Usage** | **Name** | **Literature** | **Accession ID** | **Tech. (platform)** | **Truth** | **Number of Samples** | **Number of Reference samples** | **Cell Types (Num, Type)** | **Species** |
| --- | --- | --- | --- | --- | --- | --- | --- | --- | --- |
| **Simulation** | **Jia** | Jia, et al. (2018) | PRJEB23303 | scATAC-Seq |  |  |  | 5, cardiac progenitor cells | Mouse |
| **Simulation & application** | **Corces** | Corces et al. (2016) | GSE74912 | ATAC-Seq | unknown | 42 | 79, internal | 13, hematopoietic stem cells | Human |
| **Application** |  |  | GSE74246 | RNA-Seq | unknown | 32 | 49, internal | 13, hematopoietic stem cells | Human |
| **Benchmarking  datasets** | **Shi** | MAQC (2006) | GSE5350 | Microarray | known | 60 | 60, internal | 2, universal, brain | Human |
|  | **Gong** | Gong et al. (2011) | GSE29832 | Microarray | known | 9 | 6, internal | 2, blood, breast | Human |
|  | **Shen-Orr** | Shen-Orr et al. (2010) | GSE19830 | Microarray | known | 33 | 9, internal | 3, brain, liver, lung | Rat |
|  | **Abbas** | Abbs et al. (2009) | GSE11058 | Microarray | known | 12 | 12, internal | 4, leukocytes | Human |
|  | **Kuhn** | Kuhn et al. (2011) | GSE19380 | Microarray | known | 10 | 16, internal | 4, brain | Rat |
|  | **Newman PBMC** | Newman et \. (2015) | GSE65133 | Microarray | known | 20 | 113, external | 12, leukocytes | Human |
|  | **Parsons** | Parsons et al. (2015) | PRJEB8231 | RNA-Seq | known | 30 | 9, internal | 3, brain, liver, muscle | Human |
|  | **Liu** | Liu et al. (2015) | GSE64098 | RNA-Seq | known | 24 | 16, internal | 2, adenocarcinoma | Human |

**Table S2.** Deconvolution configurations of compared algorithms. The column "Class" indicates the class that the deconvolution method belongs to; the column "Signature matrices derived" represents the source of the provided signature matrices (partial) or signature genes/peaks list (complete); column "Normalization" represents normalize method for removing batch effects, "QN" is quantile normalization, "TPM" is transcript perm million (for RNA-Seq); the column "Transformation" represents transform data methods, "Auto" is automatic transformation, including sqrt, log2, and None (without any transformation) , "-" is no transformation and "log2" is a logarithmic transformation with a base of 2.

|  | | | | **ATAC-Seq** | | **RNA-Seq** | | **Microarray** | |
| --- | --- | --- | --- | --- | --- | --- | --- | --- | --- |
| **Name** | **Literature** | **Class** | **Signature matrices (Genes/Peaks) derived** | **Normalization** | **Transformation** | **Normalization** | **Transformation** | **Normalization** | **Transformation** |
| DeconPeaker | - | partial | DeconPeaker | QN | Auto | QN | Auto | QN | Auto |
| CIBERSORT | Newman et al., 2015 |  | CIBERSORT | QN | - | QN | - | QN | - |
| dtangle | Hunt et al., 2018 |  | dtangle | TPM | log2 | TPM | log2 | QN | log2 |
| EPIC | Racle et al., 2017 |  | CIBERSORT | TPM | - | TPM | log2 | QN | log2 |
| lsfit | Abbas et al., 2009 |  |  | TPM | - | TPM | log2 | QN | - |
| qropg | Gong et al., 2011 |  |  | TPM | log2 | TPM | log2 | QN | - |
| PERT | Qiao et al., 2012 |  |  | QN | - | QN | - | QN | - |
| DeconRNASeq | Gong et al., 2013 |  |  | TPM | - | TPM | - | QN | - |
| deconf | Repsilber et al., 2010 | complete |  | TPM | - | TPM | log2 | QN | log2 |
| DSA | Zhong et al., 2013 |  |  | TPM | - | TPM | - | QN | - |

## Supplementary Figures


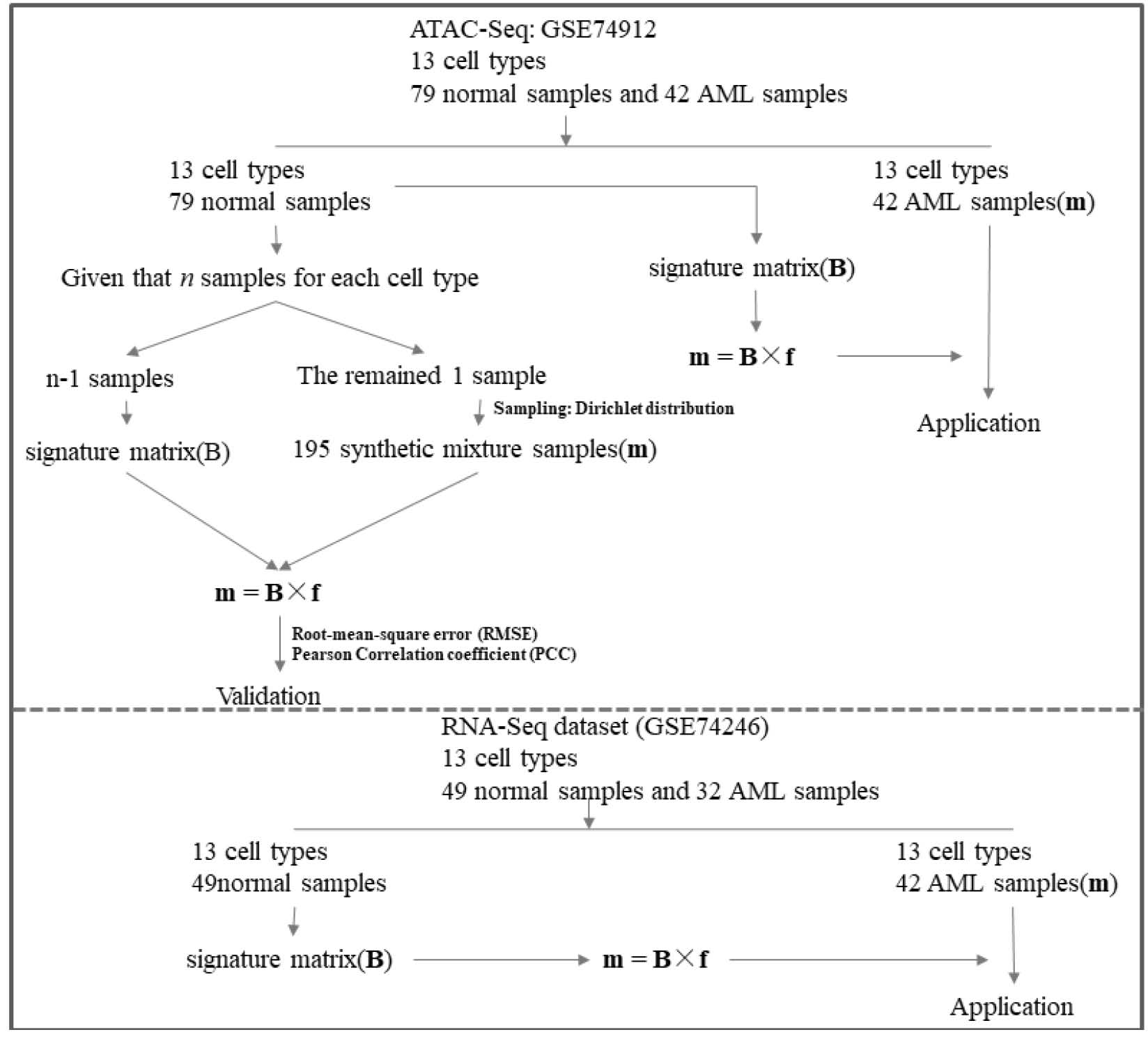


**Figure S1.** Schematic of DeconPeaker validation on ATAC-Seq synthetic mixtures and applications on AML datasets (ATAC-Seq and RNA-Seq).


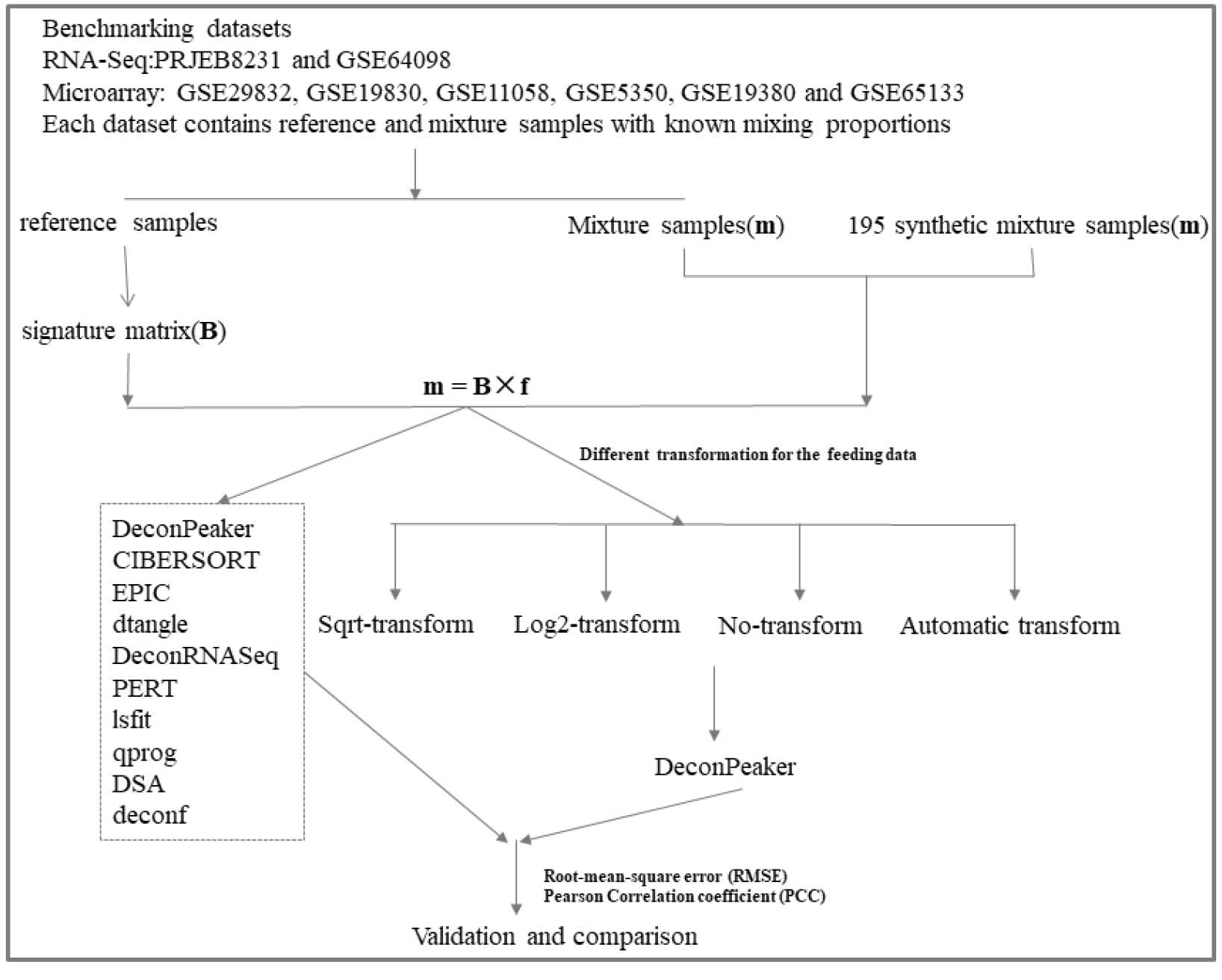


**Figure S2.** Schematic of comparisons across other known deconvolution methods and evaluation of performance of different transformations.


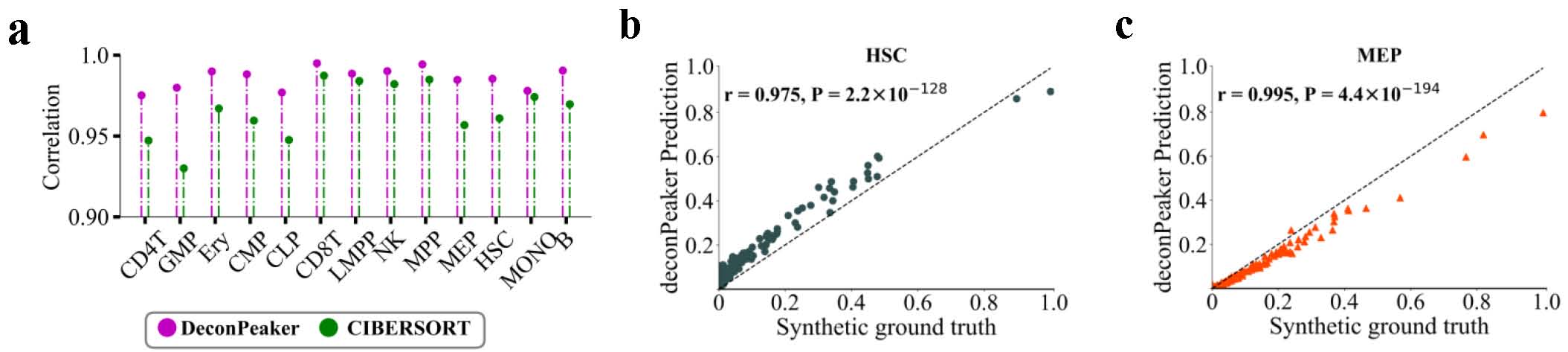


**Figure S3.** Deconvolution with DeconPeaker providing robust accuracy of most human hematopoietic cell types in the synthetic dataset. (a) Bar plots of PCC between true and estimated proportions for each cell types. (b-c) Scatter plot indicates the true proportions against the predicted proportions by DeconPeaker on the HSC (b) and MEP (c), respectively. Each point represents a specific cell type in a sample. Pearson correlation coefficient (r) was calculated between the true proportions and the prediction.

**
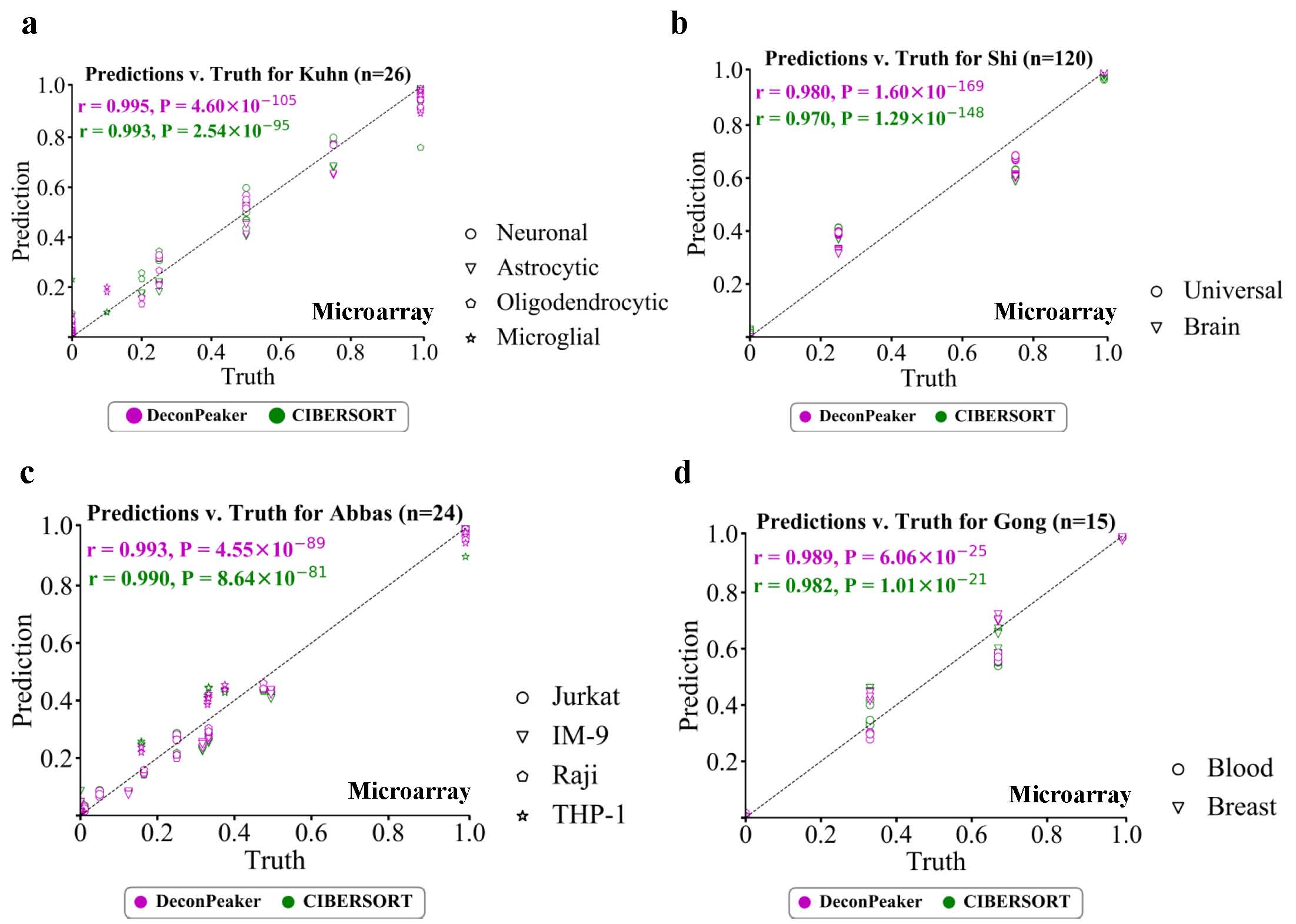
**

**Figure S4.** Performance of DeconPeaker and CIBERSORT on four microarray data sets. (a-d) Scatter plots indicate the true proportions against the predicted proportions by DeconPeaker and CIBERSORT on the four benchmarking data sets, Kuhn’s (a), Shi’s (b), Abbas’s (c) and Gong’s (d), respectively. Each point represents a specific cell type in a sample. Pearson correlation coefficient (r) was calculated between the true proportions and the prediction.


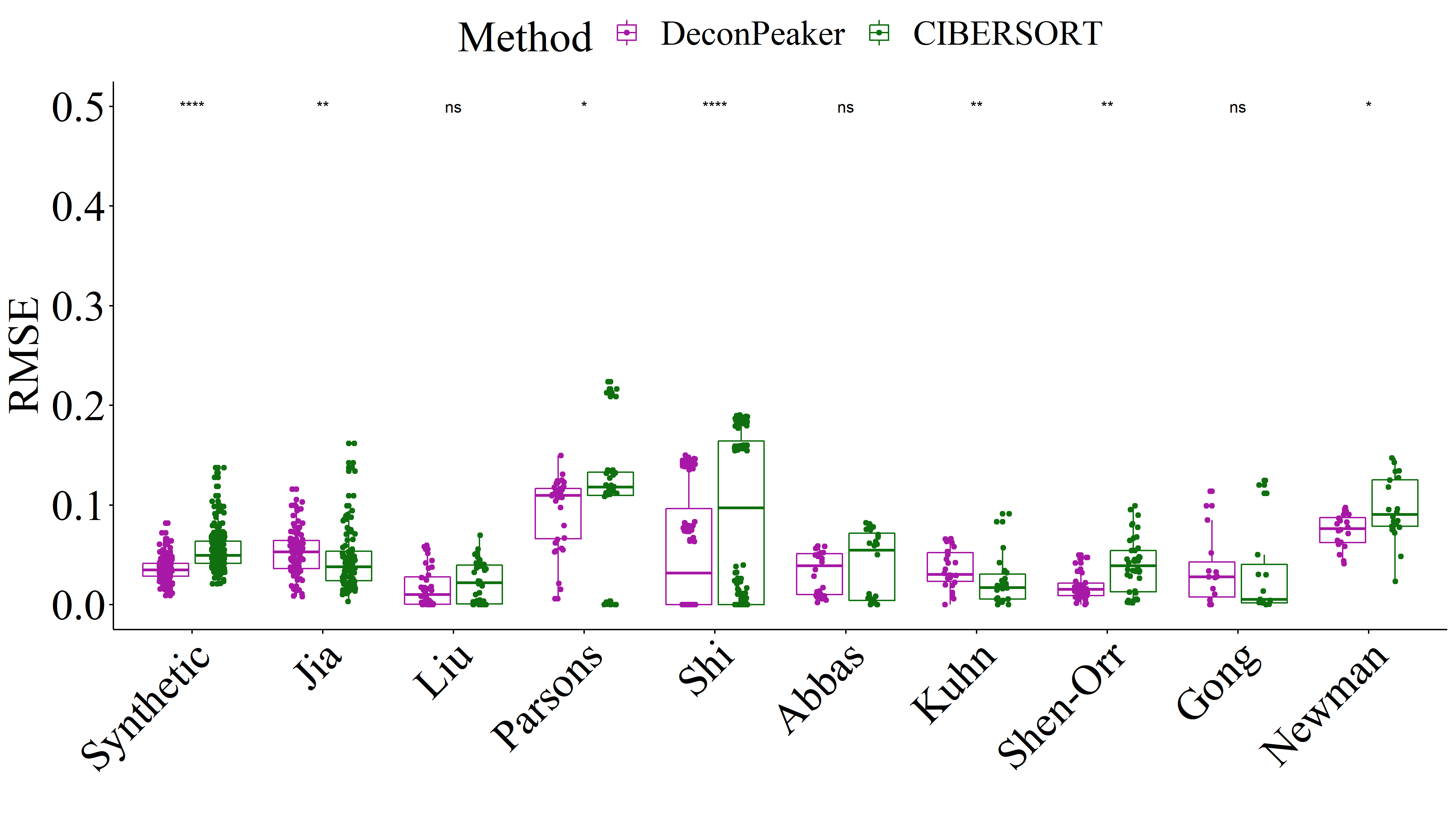


**Figure S5.** Significance test of predictions between CIBERSORT and DeconPeaker across all benchmark datasets. The thick line in the box represents the median value. The bottom and top of the boxes are the 25th and 75th percentiles (interquartile range). The whiskers encompass 1.5 times the interquartile range. The statistical difference of the two groups was compared through the Wilcoxon test. *, *P < 0.05*; **, *P < 0.01*; ***, *P < 0.001*; ****, *P < 0.0001*. Each scatter point is a specific sample.

**
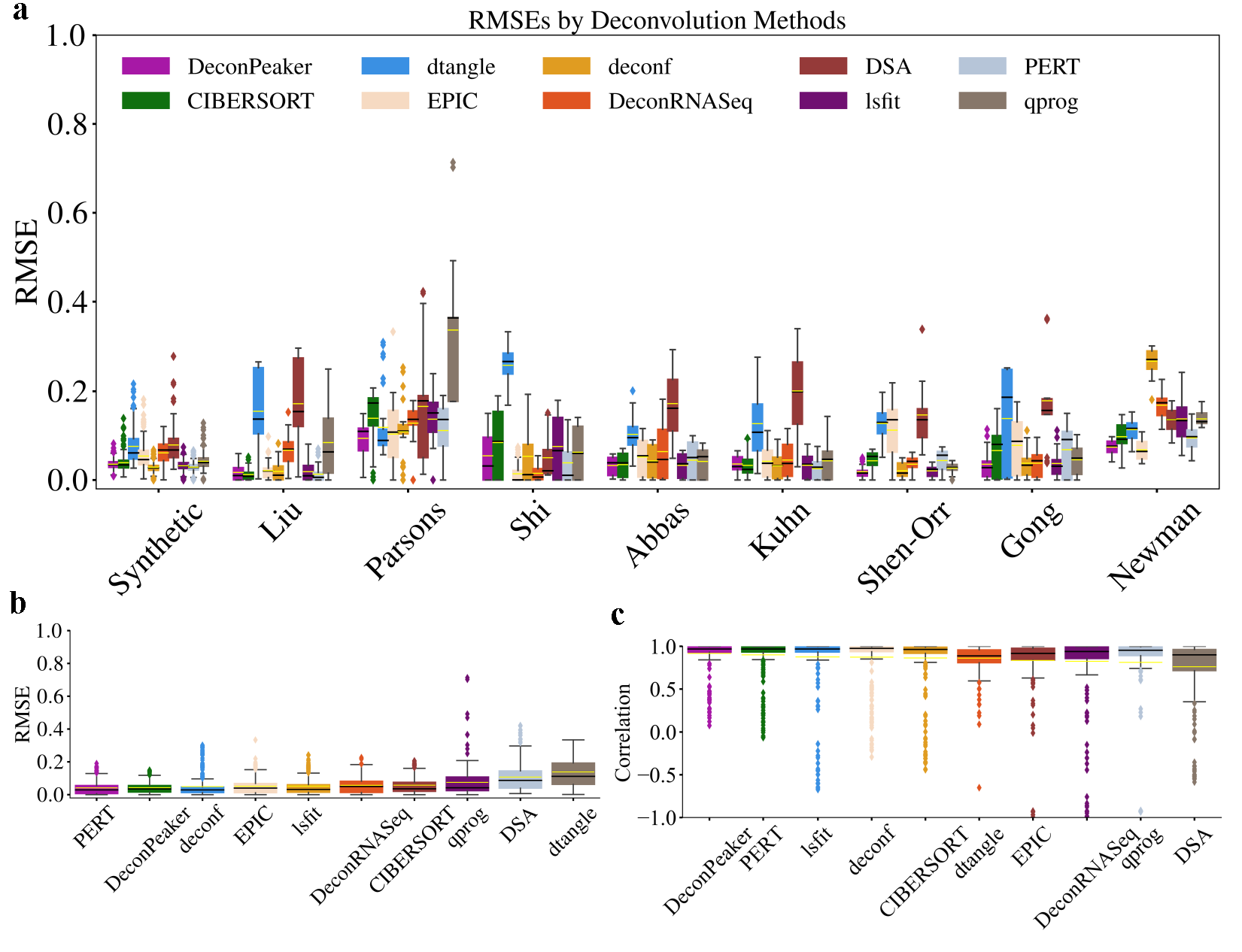
**

**Figure S6.** Comparison of DeconPeaker to other algorithms based on the same signature peaks or genes. (a) Box plots showing RMSE distribution of the predictions by algorithms on each benchmarking data set. The yellow line in each boxplot represents the average of RMSEs, while the black line is the median value. Each outlier point represents a specific mixture sample. (b) Side-by-side box plots indicating RMSEs in all benchmarking data sets. The yellow line in each boxplot represents the average of RMSEs, while the black line is the median value. From PERT to dtangle, they are sorted in ascending order based on average RMSE. Each outlier point represents a specific mixture sample. (c) Side-by-side box plots indicating correlations in all benchmarking data sets. The yellow line in each boxplot represents the average of PCCs, while the black line is the median value. From DeconPeaker to DSA, they are sorted in descending order based on average PCC. Each outlier point is a specific sample.

**
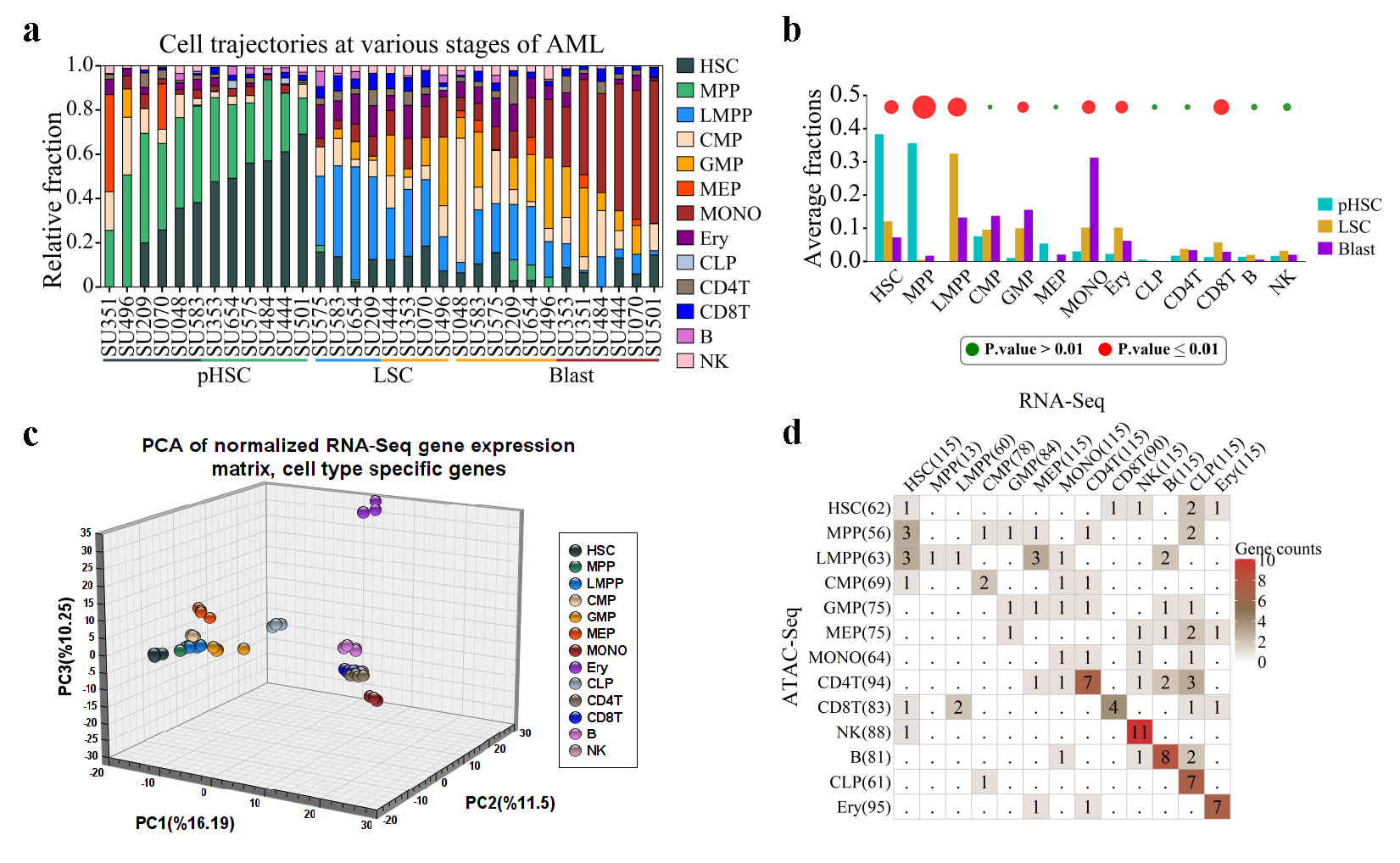
**

**Figure S7.** Deconvolution of cell composition using AML mRNA expression data. (a) Deconvolution showing the predicted contribution of various normal cell types to the transcripts landscape of AML. pHSC, LSC and Blast represent three distinct stages in the development of AML. (b) The average proportions of the predicted cell types in each stage of AML. The points above the bars indicate the statistical significance of the changes of each cell type in the three stages. Size of the point is equal to -lg*p*, where p is assessed by One-Way ANOVA test. (c) Principal Component Analysis (PCA) for genes of signature matrix. (d) Overlap between the genes associate with signature peaks from ATAC-Seq data (GSE74912) and signature genes from RNA-Seq data (GSE74246).
